# How congruent are parent reports on 3–4-year-old children’s language skills with other sources of data?

**DOI:** 10.3389/fpsyg.2023.1179999

**Published:** 2023-07-28

**Authors:** Tiia Tulviste, Astra Schults

**Affiliations:** University of Tartu, Tartu, Estonia

**Keywords:** CDI, parental reports, teacher report, language development, communicative development, parental education, Estonian

## Abstract

**Background:**

Parental report measures such as the MacArthur-Bates Communicative Development Inventories (CDIs) are frequently used to study communicative skills of children under 3 years of age. Less is known about the usability of such reports for assessing communication skills in older children due to their advanced language skills, and a higher variety of communicative partners and communication contexts.

**Aims:**

To assess the concurrent and predictive validity of the Estonian (E) CDI-III at ages 3;0 and 4;0 years. The first research goal was to examine its concurrent variability—associations with teacher reports and directly measured language skills. The second goal of the study was to investigate the predictive validity of parent reports—the degree to which parent-and teacher-reported language scores for children at age 3;0 are useful for predicting examiner-administered language comprehension and production scores 1 year later.

**Methods:**

Estonian monolingual children were investigated longitudinally at ages 3;0 (*n* = 104; M age = 35.77 months, *SD =* 0.84; 42% males) and 4;0 (*n* = 87; M age = 48.18 months, *SD =* 1.16; 42% males) years. Children were assessed with the parent-reported ECDI-III, with teacher-reported assessments on children’s talkativeness, vocabulary size and grammatical skills, and the examiner-administered New Reynell Developmental Language Scales IV (NRDLS).

**Results:**

Results indicated significant positive relationships between the ECDI-III total scores, teacher reports, and directly measured language comprehension and production scores, demonstrating concurrent validity of parental reports of children language skills at both ages. When controlling for mothers’ education, children’s gender, and reported language difficulties, parental and teacher reports were predictive of language production scores, whereas only parental reports predicted comprehension scores 1 year later. None of the controls was predictive of later language comprehension and production scores.

**Conclusion:**

In sum, good concurrent and predictive validity of the ECDI-III shows that the instrument is a valid tool for assessing communicative skills in Estonian children. Results suggest that parent reports can offer useable information also about communicative skills of children older than three years.

## 1. Introduction

Parental report measures such as the MacArthur-Bates Communicative Development Inventories (MB-CDIs) are widely used instruments for estimating language skills of infants and toddlers (Fenson et al., 2007). Parent reports are time-and cost-effective in obtaining a picture of child early development and allowing to gather data on large samples. Unlike direct testing, parent report instruments do not require a well-trained estimator, and children do not need to communicate with an unfamiliar adult and to solve tasks that may be decontextualized and novel for them (Fenson et al., 2007). Parents are good reporters on children’s language skills likely due to the possibility of observing children communicating in various situations and knowing what children are able to say. Their reports are not influenced by the child’s current mood, health, attention state or temperament (e.g., shyness) like direct assessment. Being their children’s first teachers, parents stimulate their development, and CDIs could serve as a tool for monitoring children’s language learning. Because formal testing is difficult to conduct in small children, parent reports are especially suitable for estimation of communicative abilities below 3 years of age (Fenson et al., 2007).

A number of studies show the utility, validity, and reliability of parent reports on infants’ and toddlers’ language skills (see Fenson et al., 2007; Law and Roy, 2008 for reviews). Significant correlations have been found between parental reports, concurrent spontaneous speech measures and direct assessments of child language skills (Pan et al., 2004). Recent studies show strong predictive validity of parents reports on children’s early language skills. For example, Bleses et al. (2016) indicated that early expressive vocabulary predicts reading and math outcomes 10 years later. Less is known about parental reports as a source of information about older children’s language skills. Unlike younger children, they have better communicative skills.

There is a growing body of studies addressing the utility and validity of parent reports for assessing communication skills in children over 3 years of age (Dionne et al., 2003; Eriksson, 2017). Several adaptations of the CDI-III have been developed based on the original version of the instrument (Dionne et al., 2003), for example for Basque, Norwegian, and Spanish (see Kas et al., 2022). Studies using the CDI-III original version reflect to the ability of parents to provide valid estimation of children’s language skills also at ages 30–37 month of age (Fenson et al., 2007). Validation studies have found ceiling effects in “Syntactic complexity” and “Uses of language” subscales of the original version after 33 months (Fenson et al., 2007) and in all subscales of the Basque version after 42 months (see Kas et al., 2022). Other adaptations have been used the Swedish version of the CDI-III (Eriksson, 2017) designed for children from 30 months to 48 months, as for example Estonian (Tulviste and Schults, 2020), Hungarian (Kas et al., 2022) and Portuguese CDI-IIIs (Cadime et al., 2021). The Swedish version covers a longer period of time than the original version of the CDI-III. Eriksson (2017) found a slight ceiling effect in the syntactic complexity and metalinguistic awareness subscales after 45 months.

All versions of the CDI-IIIs are relatively new and only a few studies have focused on its concurrent validity. Odeskog and Stenberg reported low correlations between the Swedish CDI-III and the Peabody Picture Vocabulary Test and the Boston Naming Test in 44 children aged 36–47 months (see Kas et al., 2022). Tulviste and Schults (2020) found medium correlations between the vocabulary scores of the ECDI-III and directly measured Reynell Language Comprehension and Production Scale scores in 100 children at the age of 3 years. Cadime et al. (2021) showed moderate correlations with the Vocabulary total score and the Syntax score of the European Portuguese CDI-III and the language score of the Griffiths Mental Development Scales in 23 children aged 30–48 months. Studies using standardized tests to investigate the concurrent validity of CDI-IIIs suffer from limited age range and small sample size. There is a lack of studies on the predictive validity of CDI-IIIs. Thus, more studies are needed to address the concurrent and predictive validity of parent report in children aged 3;0 years and older.

Given that nowadays most children beyond 3;0 years of age are enrolled in kindergarten and spend long days in child-care settings, kindergarten teachers also play an important role in facilitating child development. Teachers are expected to monitor child language acquisition and identify children with speech and communication problems, because early intervention is more efficient than later intervention (e.g., Dale et al., 2003). Accordingly, some researchers have started to use kindergarten teachers as a source of information about children’s language skills via CDIs (Vagh et al., 2009; Bleses et al., 2018; Cadime et al., 2021). Teachers are seen as good judges of child language abilities, working frequently with groups of same-age children. That provides them plenty of opportunities to compare communicative abilities of children in similar age. Kindergarten teachers also have the opportunity to observe children interacting with different communicative partners in different interactional contexts, despite the range of contexts being limited. Moreover, during teacher training, they have studied child development milestones, including their communicative development, and how to stimulate child development.

Some authors suggest to use multiple reporters for estimating children’s language skills, considering the possibility that children may talk about somewhat different topics with different conversational partners in and outside home, and in case of bilingual children, also involve different languages. Many parents of bilingual children may not be able to report children’s non-native language abilities (Vagh et al., 2009). However, De Houwer et al. (2005) found in a study with monolingual children that although there are significant correlations among estimations about children’s language skills done by different reporters (mothers, fathers and the third person) via CDI, they assess language skills of the same child rather differently, especially in case of older children whose language skills are relatively high.

Thus, there are some concerns about the use of reports with older children due to their increased communicative abilities as well as a higher number of conversational partners and interactional contexts. Therefore, studies providing more information about the utility of using reports as a source of information about children’s language skills after 3 years, are needed. The current study assessed the concurrent and predictive validity of the ECDI-III – the Estonian adaption of the Swedish CDI III (Eriksson, 2017), using the data gathered at two timepoints: at children’s age of 3;0 and 4;0 years.

The first aim of the present study was to test the concurrent validity of parent reports on general communicative skills (the total score of the ECDI-III) as compared with teacher reports, and directly assessed 3-and 4-year-old children’s language comprehension and production scores by a standardized examiner-administered language assessment New Reynell Developmental Language Scales (NRDLS) (Edwards et al., 2011). Although CDIs have been used to assess various aspects of child early language, most previous studies have focused on infants’ and toddlers’ vocabulary skills and checked the validity and reliability of the vocabulary list (Pan et al., 2004; Fenson et al., 2007; Tulviste and Schults, 2020). Some other studies have explored both vocabulary and grammatical development, since multiword sentences, basic sentence structure and inflections of the native language are also good indicators of the rate of language development (Fenson et al., 2007). The children participating in our study were at ages 3;0 and 4;0 years when grammatical and phonological skills are also indicative about the level of their language skills. Therefore, in addition to vocabulary scores, we also used scores from other subscales, and calculated total ECDI-III scores to serve as an indicator of more general language skills of the child. Another reason for using total scores instead of only vocabulary was that the NRDLS assesses general language comprehension and production skills. As teacher reports we used compound teacher ratings of their answers to three questions about children’s communicative abilities: teachers’ evaluations of child’s talkativeness, size of vocabulary, and complexity of sentences compared to the child’s age mates.

The second aim of the study was to investigate to what extent parent-and teacher reported language skills have substantial predictive validity, evaluating the utility of both sources of reports around the time of their third birthday for predicting language skills around their 4th birthday. To explore how well parent and teacher reports predict future language skills, we also considered parental education and child gender, established as important predictors of child language development (Fenson et al., 2007). Moreover, education might also affect how adequate the reports are. As pointed out by Stiles (1994), the CDIs place high demands on parents to reflect on different aspects of child communication. It is likely that parents with higher educational level manage better in filling out the questionnaire as they have better knowledge about what children are able to say (Fenson et al., 2007). Plenty of studies mostly based on parental reports have found gender differences in children’s communicative skills. Girls have demonstrated to have larger vocabularies and quicker rates of grammatical development than boys (Bornstein et al., 2004; Eriksson et al., 2012; Simonsen et al., 2014; Urm and Tulviste, 2016).

Thus, the study addressed the following questions:

1. Are parent reports at ages 3;0 and 4;0 valid estimators of Estonian children’s language skills when compared with teacher reports and experimenter-measured language skills (language comprehension and production via the NRDLS)?
2. To what extent do earlier parent and teacher reports predict children’s language skills 1 year later, when controlling for mother’s education and child’s gender?

## 2. Methods

### 2.1. Participants

As part of a larger research project, „The role of early social contexts in supporting the development of language skills: A way to close the academic achievement gap“, led by the first author of the current paper, a longitudinal study to validate ECDI-III was carried out. The first gathering of data was around children’s third birthday for 104 children (44 boys, 60 girls, age range from 2;10 to 3;3, *M* = 35.77 months, *SD* = 0.84). The second gathering of data was around children’s fourth birthday (*M* = 48.18, *SD =* 1.16, age range from 3;10 to 4;2) for 87 of the original participants. At first gathering of data 20 children (12 boys and 8 girls) were identified by their parents as experiencing difficulties with language development, 17 of them (9 boys and 8 girls) participated also at the second gathering of data. According to the parents, the children were otherwise healthy. According to parental reports, Estonian was the dominant language in the families, although 12 children had a parent or grandparents who sometimes (less often than daily, for a couple of hours at a time) spoke another language with the child. None of the participants were excluded due to reported difficulties with language development nor due to exposure to another language. Most participants were from middle or higher SES homes with mothers having completed upper secondary (33%) or university (54%) education, 7% of the participants had parents with a lower secondary education, and parental education data were not available for 6% of children. The income for the family was more than one average wage for 82% of the participants. One hundred and one of the participating children were attending kindergarten or a playgroup regularly.

### 2.2. Procedure

Participants were recruited through kindergartens and child care centers. We sent an invitation to participate to all kindergartens and child care centers in the cities of Tartu and Pärnu, Estonia, where there were groups for 3-year-olds. If the head of the institution agreed to take part in the study, they asked the teachers of three-year-olds to hand out the invitations to the families. An invitation to participate was sent shortly before the child’s third birthday to 207 families. Roughly half of the invited families agreed to participate, signing the informed consent form. The teachers who had children from their group participating in the study were asked to fill in Social Skills Questionnaires (Häidkind et al., 2018) on paper. As there were two teachers per group, they decided themselves which one of them would fill in the Social Skills Questionnaire for each participating child. Most of the teachers filled in one or two questionnaires, maximum number of Social Skills Questionnaires filled in by one teacher was four. Trained research assistants visited families at home on two occasions (around child’s third and fourth birthday) and administered the Estonian version of NRDLS (Edwards et al., 2011), first the Comprehension Scale and then the Production Scale. If a child did not comply to take both scales of the NRDLS during one visit (e.g., being fussy, tired), the assistants visited the family again. Five children did not comply the Production Scale during the second visit either, and NRDLS was left uncompleted. At both visits, the assistants asked the parents to complete the questionnaires (subject information sheet, ECDI-III, and Social Skills Questionnaire) within the next couple of days. The parents could choose if they preferred to fill in the questionnaires online or on paper. A paper version of the questionnaires was handed out to them with a prepaid return envelope. Seventy eight of the parents completed the questionnaires online and 26 on paper. We sent gentle reminders about the questionnaires waiting to be completed to those families who had agreed to participate in the study but who had not completed the questionnaires in 2 weeks after having received either the link to the questionnaire or the questionnaire on paper. Still, five parents completed only the vocabulary section of the ECDI-III. Written feedback on the child’s language results was sent to the parents. Day-care teachers who provided reports on children’s communicative skills were provided gift cards for their help, as were families who participated at both times of data gathering. Children received stickers as presents.

The Research Ethics Committee of the University of Tartu approved the study. The CDI Advisory Board approved the development of adaptations of CDI-III to Estonian, based on the work already authorized and done for Swedish.

### 2.3. Materials

#### 2.3.1. ECDI-III

The ECDI-III (Tulviste and Schults, 2020) is the Estonian adaptation of the CDI-III developed for Swedish by Eriksson (2017), designed for children 30 to 48 months old and consists of 6 subscales. First, in the level of communication section parents have to indicate if their child can speak and how complex their child’s speech is (6 alternative items). The parents are asked to continue with filling in the rest of the checklist only if they have marked an alternative indicating that their child uses at least one-word utterances.

Second, in a 100 item vocabulary list the parents have to indicate words (from the list of 100 words, mainly verbs and adjectives) that their child produces in four themes: food words (16 items), body words (26 items), mental words (30 items), and emotion words (28 items).

Third, in the syntax section the parents are asked about their child’s grammar usage and sentence complexity. Grammar usage lists 7 items including the plural, comparisons, past tense, and conjunctions. The parents are asked to indicate for each item if their child has never used a particular example of grammar (scored 0), has used it several times (scored 1), or uses it on a daily basis (scored 2). Thus, the possible score for grammar usage ranged from 0 to 14. Sentence complexity consists of 10 pairs of sentences that consists of a short sentence with simple grammar and a complex, more elaborated sentence, both expressing the same main meaning. Regarding the pairs of simple and complex sentences the parents had to indicate for each pair if their child currently uses the simpler one (scored 0), alternates between simple and complex sentences (scored 1), or currently uses the more complex one (scored 2). The maximum score of sentence complexity is 20. The maximum score for syntax section is 34.

Fourth, in the metalinguistic awareness section the parents assess phonological awareness and orthographic awareness of the children. For phonological awareness (3 items), the parents have to indicate whether their child is able (scored 1) or unable (scored 0) to notice rhymes, to break words into syllables, and to understand that some people speak a foreign language. For orthographic awareness (4 items), the parents have to indicate whether their child is engaged in activities related to letters (scored 1 or 0 respectively) such as being interested in letters, recognizing some letters, writing some letters, and writing some short familiar words. The maximum score for metalinguistic awareness is seven.

Fifth, in the pronunciation section the parents are asked how their child’s speech sounds compared to other children of the same age, and if their child has pronunciation difficulties. For five of the listed items parents are asked to indicate if their child has difficulties (scored 0) or not (scored 1) with the pronunciation of more difficult phonemes (*r*-sound and *s*-sound), changing the form of words as they are produced, and if strangers are able to understand the child. The final item asked if the child’s speech resembles that of a younger child (scored 0), an age mate (scored 1), or an older child (scored 2). The maximum score for the pronunciation section is seven. All subscale scores were summed (max = 154).

#### 2.3.2. The New Reynell developmental language scales

Children’s language comprehension and production skills were tested using NRDLS (Edwards et al., 2011). This is the most recent version of the well-known structured tests—the Reynell Developmental Language Scales. The scales test vocabulary and grammar: the comprehension and production of single words (nouns and verbs) as well as of simple and complex sentences with easiest items at the beginning and most difficult in the end. Objects, pictures and variety of testing procedures are used to maintain the attention of children. First the comprehension tasks and then the production tasks were administered to each child individually by a research assistant during home visits. The Comprehension Scale consists of 72 items and the Production Scale of 64 items. An adapted version for Estonian children has the same number of items in both scales, but wording of some items in the pronouns, complex sentences, and grammatical judgment sections have been changed because of language differences between Estonian and English. Estonian is an agglutinative language, characterized by a large number of cases (14 productive cases), no grammatical gender (either of nouns or personal pronouns), and no articles. In the Estonian pronouns section, *ennast* “himself/herself” and *teda* “him/her” have been used. The complex sentences section assesses the child’s comprehension of passive sentences, and the thematic roles expressed by the passive sentences are reversable. The child is expected to show the picture that goes with what is said, e.g., to show the picture of a baby being fed by the mother after the experimenter said “The mother is fed by the baby.” Because these passive sentences from the original English versions were not translatable into Estonian, sentences in the active voice (e.g., “Tita annab emale süüa”) are used and the child has to work out who is doing what to whom. The study has preliminary norms only for 3–4-year-old children based on 255 children in the age range from 34 to 50 months (Tulviste, unpublished data). In the present study, Cronbach’s alpha was used to access the internal consistency of items within the scales. These were 0.93 for the Comprehension scale and 0.96 for the Production scale. At both ages, the two scales correlated highly, *r* = 0.74 as the children were three and *r* = 0.84 as the children were four.

#### 2.3.3. Teacher reports

From Social Skills Questionnaire (Häidkind et al., 2018) we included three items to the analyses. Social Skills Questionnaire (SSQ) is based on social skills classification (Merrell and Gimpel, 1998) as well as on the Estonian curriculum of preschool childcare institutions. The questionnaire is designed to be filled in by the kindergarten or playgroup teachers who have many opportunities to observe the children in everyday social situations. Three items from Social Skills Questionnaire included to this study were teachers’ evaluations of child’s talkativeness, size of vocabulary, and complexity of sentences compared to the child’s age mates at the first data collection. These evaluations were included in the analyses as these give an indication for teachers’ experience with child’s language production. The evaluations were given for each of the items as 1 point if the child was at a lower level, 2 points if the child was on bar, and 3 points if the child was at a higher level compared to the age mates. As each of these items was positively correlated with the other two (*r*s = 0.51 to 0.80) we combined the scores of these three into one sum showing teacher’s general evaluation of child’s language skills.

#### 2.3.4. Data analysis

All the answers given by the parents in ECDI III and teachers in SSQ as well as NRDLS test results for the children were included in the data set. Two multiple regression analyses were conducted to determine the extent to which the ECDI-III total score and teacher reports at age 3;0 predict comprehension and production scores at age 4;0, controlling for maternal education (with vs. without higher education), child gender and reported language difficulties (with vs. without language difficulties).

## 3. Results

### 3.1. Internal consistency of ECDI-III

Cronbach’s α for the whole list of words was *α* = 0.97 (standardized *α* = *NA*) as the children were 3 years old. As the children were four there were too many items with null variance to calculate the Cronbach’s α for the whole list of words. Cronbach’s α for the syntax section were *α* = 0.92 (standardized *α* = 0.92) both as the children were three and four, pronunciation accuracy as the children were three α = 0.72 (standardized *α* = 0.71) and as the children were four *α* = 0.75 (standardized *α* = 0.75), metalinguistic awareness as the children were three α = 0.66 (standardized *α* = 0.63) and as the children were four *α* = 0.57 (standardized *α* = 0.57).

### 3.2. Variability in children’s language measures at both data collection times

As shown in Table 1, children’s language skills varied greatly at both time points, regardless of the assessment tool used. Furthermore, in 1 year all language scores central to the study increased significantly.

**Table 1:** Descriptive statistics of language measures and differences in scores from two data collections.

	First data collection at age 3;0					Second data collection at age 4;0				*t*
	*N*	*M*	*SD*	Range		*N*	*M*	*SD*	Range	
ECDI-III										
Syntax	99	17.52	8.71	0–34		78	24.56	7.20	0–34	11.48
Pronunciation	98	3.85	1.91	0–7		79	4.38	2.10	0–7	4.40
Metalinguistic skills	99	2.33	1.68	0–6		79	4.33	1.65	0–7	12.90
Vocabulary	103	52.83	21.82	0–92		79	73.18	17.91	12–100	13.40
Total	98	83.94	29.05	12–138		78	113.01	24.24	27–151	16.31
NRDLS										
Comprehension	99	48.74	10.87	9–67		87	58.92	8.99	27–72	13.08
Productive	94	33.03	13.32	2–61		87	46.72	12.20	9–64	14.91
Total	94	83.00	21.05	28–128		87	105.64	20.33	39–136	17.24
Teacher report	84	6.26	2.21	3–9						

*T*-tests on each subscale and scale were significant at *p* < 0.001.

#### 3.2.1. ECDI-III scores

##### 3.2.1.1. Level of communication

Around the third birthday three of the 104 participants were reported by the parents as not yet producing one-word utterances. At the same time 14 of the children were using short utterances and 86 were using sentences. A year later all of the 87 participants were reported by their parents to be using at least one-word utterances. Four of them were using short utterances and 75 were using sentences. Descriptive statistics of the subscales (Vocabulary, Syntax, Metalinguistic skills, Pronunciation) and the total scores of the ECDI-III at two data gatherings are presented in Table 1.

#### 3.2.2. The New Reynell developmental language scales scores

The maximum score for language comprehension scale was 72, for language production scale 64, and total maximums score was 136. At 3 years of age the average score for language comprehension was around 49, the average score for language production was 33, and the average total score was 83. At 4 years of age the average score for language comprehension had increased for 10 points, being 59, the average score for language production had increased for 13 points, being 46, and the average total score had increased for 20 points, being 105.

#### 3.2.3. Teacher reports

As the children were 3 years old, we asked for teachers’ evaluations of child’s talkativeness, size of vocabulary, and complexity of sentences compared to the child’s age mates with resulting maximum score being 9. At the age of three average score of teachers’ evaluations was around 6. See Table 2 for descriptive statistics and for differences between scores from two data collections.

**Table 2:** Correlations of language measurements at both data collections.

	First data collection at age 3;0									Second data collection at age 4;0						
	Syntax	Pron	Meta	Vocab	Total	Compr	Prod	Total		Syntax	Pron	Meta	Vocab	Total	Compr	Prod
1st data collection																
ECDI-III																
Pron	**0.53**															
Meta	**0.32**	*0.19*														
Vocab	**0.70**	**0.39**	**0.38**													
Total	**0.85**	**0.52**	**0.44**	**0.97**												
NRDLS																
Compr	**0.61**	**0.40**	**0.45**	**0.63**	**0.69**											
Prod	**0.61**	**0.50**	**0.37**	**0.62**	**0.68**	**0.74**										
	**0.63**	**0.49**	**0.43**	**0.67**	**0.73**	**0.91**	**0.96**									
TE	**0.47**	**0.51**	*0.13*	**0.42**	**0.46**	**0.43**	**0.39**	**0.41**								
2nd data collection																
ECDI-III																
Syntax	**0.74**	**0.46**	**0.22**	**0.67**	**0.73**	**0.59**	**0.63**	**0.60**	**0.44**							
Pron	**0.45**	**0.73**	*0.13*	**0.45**	**0.49**	**0.39**	**0.39**	**0.39**	**0.51**	**0.56**						
Meta	**0.37**	**0.24**	**0.63**	**0.51**	**0.46**	**0.40**	**0.33**	**0.36**	**0.29**	**0.39**	**0.40**					
Vocab	**0.49**	**0.27**	**0.31**	**0.75**	**0.69**	**0.54**	**0.48**	**0.50**	**0.45**	**0.68**	**0.44**	**0.43**				
Total	**0.65**	**0.42**	**0.34**	**0.77**	**0.79**	**0.62**	**0.60**	**0.61**	**0.48**	**0.85**	**0.56**	**0.48**	**0.96**			
NRDLS																
Compr	**0.66**	**0.39**	**0.34**	**0.67**	**0.67**	**0.77**	**0.66**	**0.73**	**0.48**	**0.64**	**0.45**	**0.47**	**0.66**	**0.68**		
Prod	**0.59**	**0.42**	**0.33**	**0.65**	**0.63**	**0.71**	**0.77**	**0.79**	**0.58**	**0.60**	**0.52**	**0.50**	**0.62**	**0.64**	**0.84**	
Total	**0.65**	**0.43**	**0.35**	**0.69**	**0.68**	**0.77**	**0.76**	**0.81**	**0.56**	**0.65**	**0.51**	**0.51**	**0.66**	**0.69**	**0.95**	**0.97**

All statistically significant correlations at *p* < 0.05 are in bold. Pron, Pronunciation; Meta, Metalinguistic skills; TE, Teacher report; Compr, Comprehension; Prod, Production; Vocab, Vocabulary; Total, RCDI-III Total.

### 3.3. Relations between the language measures at both data collections

The correlations for all of the language measures at both ages and between two ages are presented in Table 2. The significant positive correlations of all of the language measures between the first and the second data collection ranged from *r* = 0.22 to *r* = 0.81, *p* < 0.05. The only correlations that were not significant were between metalinguistic awareness at 3 years of age and pronunciation at 4 years of age.

The significant correlations of parental reports of syntax, pronunciation, metalinguistic awareness, and vocabulary with teacher reports of child’s language skills ranged from *r* = 0.41 to *r* = 0.51, *p* < 0.05 as the children were 3 years of age. The only correlation that was not significant was between metalinguistic awareness and the teacher report of child’s language skills at that age. The correlations of parental reports of syntax, pronunciation, metalinguistic awareness, and vocabulary from the second time of data collection with teacher reports of child’s language skills ranged from *r* = 0.29 to *r* = 0.58, *p* < 0.05.

The correlations of ECDI-III subscales with NRLDS subscales ranged from *r* = 0.37 to *r* = 0.69, *p* < 0.05 as the children were 3 years old, from *r* = 0.45 to *r* = 0.66, *p* < 0.05 as the children were 4 years old, and from *r* = 0.33 to *r* = 0.67, *p* < 0.05 between two ages. The correlations of ECDI-III and NRLDS total scores were *r* = 0.73 to, *p* < 0.05 at 3 years of age, *r* = 0.69, *p* < 0.05 at 4 years of age, and *r* = 0.68, *p* < 0.05 between two ages.

### 3.4. Mother and teacher reports as predictors of later language skills

In Tables 3, 4 we provide findings from two separate multiple regression analyses showing to what extent the ECDI-III total scores and teacher reports at age 3;0 predict children’s comprehension and production scores measured by the NRDLS at age 4;0, controlling for mothers’ education, child gender, and reported language difficulties. In both analyses, we entered the ECDI-III total score first (Model 1). Then we explored teacher reports as the predictor (Model 2). Next, we entered the ECDI-III total score and teacher reports in one model to investigate their combined effect (Model 3). Then we added mothers’ education (Model 4), and finally in Model 5 also child gender and reported language difficulties. As shown in Table 3, ECDI-III total score alone explains approximately 45%, and teacher reports alone 23% of the variance in comprehension scores. Together they explained 49% of the variance. When adding mothers’ education, the R-squared statistics increases 2%, and only the ECDI-III total score remained the significant predictor. When adding child gender and reported language difficulties only the ECDI-III total score remained the significant predictor. Neither teacher reports nor the control predicted comprehension scores significantly.

**Table 3:** Regression models predicting children’s comprehension scores at age 4;0 (NRDLS) on the basis of child language measures at age 3;0.

Predictors	NRDLS comprehension β-coefficient (standard error)				
	Model 1	Model 2	Model 3	Model 4	Model 5
Intercept	41.50*** (2.27)	46.64*** (2.86)	38.47*** (2.74)	38.56*** (2.70)	38.06*** (3.77)
ECDI total	0.21*** (0.03)		0.18*** (0.03)	0.17*** (0.03)	0.17*** (0.03)
Teacher report		1.96*** (0.43)	0.90* (0.40)	0.75 (0.40)	0.64 (0.44)
Maternal education^a^				2.91 (1.67)	2.85 (1.70)
Gender					0.91 (1.66)
Reported language difficulties^b^					−0.68 (2.22)
*R* ^2^	0.45	0.23	0.49	0.51	0.52
*F*	65.54***	20.63***	32.14***	23.09***	13.60***

ECDI-III—ECDI-III total score. *p < 0.05, ***p < 0.001. ^a^Maternal education was represented as a dummy variable with no university education as the reference category. ^b^Child’s language difficulties was represented as a dummy variable with no language difficulties serving as the reference category.

**Table 4:** Regression models predicting children’s productive scores at age 4;0 (NRDLS) on the basis of child language measures at age 3;0.

Predictors	NRDLS production β-coefficient (standard error)				
	Model 1	Model 2	Model 3	Model 4	Model 5
Intercept	24.60*** (3.24)	26.72*** (3.62)	17.77*** (3.67)	17.82*** (3.69)	16.64*** (5.09)
ECDI-III	0.26*** (0.04)		0.19*** (0.04)	0.19*** (0.04)	0.18*** (0.04)
Teacher report		3.19*** (0.55)	2.04*** (0.53)	1.95*** (0.55)	1.67** (0.60)
Maternal education^a^				1.59 (2.28)	1.40 (2.31)
Gender					2.38 (2.25)
Reported language difficulties^b^					−1.96 (3.00)
*R^2^*	0.39	0.34	0.50	0.54	0.52
*F*	52.14***	34.33***	33.87***	26.15***	13.79***

ECDI-III—ECDI-III total score. **p < 0.01, ***p < 0.001. ^a^Maternal education was represented as a dummy variable with no university education as the reference category. ^b^Child’s language difficulties was represented as a dummy variable with no language difficulties serving as the reference category.

As shown in Table 4, the ECDI-III total alone predicted 39%, and teacher reports alone 35% of the variability in production scores. When combined they predicted 50% of the variance in production scores and both remained significant predictors. When mother education was added in model already containing the ECDI-III total score and teacher reports (Model 3), the R-squared statistic increased to 54%, but mother education was not a significant predictor. Adding child gender and reported language difficulties decreased approximately 2% the predictability of production scores, the ECDI-III and teacher reports remained predictive. None of the controls was predictive of production scores at age 4;0.

## 4. Discussion

Numerous studies have demonstrated that early language skills are good indicators of children’s concurrent and future development and adjustment. However, some researchers have questioned the concurrent and predictive validity of parent report measures as assessment tools of language skills of children after their first years of life, because with growing age children become more communicative, having more conversational partners and interactional contexts than during their first years of life. Therefore, the present study sets out to compare parent reports with two other sources of information about Estonian children’s language skills—teacher reports and experimenter assessments—at ages 3;0 and 4;0 years.

### 4.1. Concurrent correlations between the ECDI-III, teacher reports and directly measured language scores

The first aim of the study was to explore the concurrent validity and utility of report measures in estimating child language skills at ages 3;0 and 4;0, taking measures of language comprehension and production administered by an expert examiner via the NRDLS as a golden standard. Results of correlational analysis indicated significant positive correlations of acceptable magnitude (*r*s = 0.64–0.69) between the ECDI-III total scores and with directly measured language comprehension and production scores. The strongest correlations of directly measured language comprehension and production scores were with vocabulary and syntax scores of the ECDI-III. The finding suggested that vocabulary and grammar development were the most indicative CDI measures of children’s language skills also in the age period studied in our study. Other aspects of language development (i.e., pronunciation and metalinguistic abilities) provided only some additional information. The results are in line with most validation studies with younger children, where only the vocabulary list or in some studies vocabulary and grammar sections were addressed (Fenson et al., 2007).

The study found lower and moderate (*r*s = 0.37–0.44 at Wave 1, and *r*s = 0.48–0.58 at Wave 2), albeit significant correlations between scores reported by teachers and those obtained by parent report or direct assessments. Correlations of teacher reports with direct language measures were lower than those of parent reports, likely in part, since teachers were asked only 3 questions – to estimate child’s talkativeness, size of vocabulary, and complexity of sentences compared to the child’s age mates. A reason for low correlations between two report measures may also lie in good communicative abilities of children at this age that makes it difficult for a reporter to capture all of what children are able to say. Moreover, parents and teachers observe children communicating in different interactional contexts and with different communicative partners (De Houwer et al., 2005). Keeping this in mind, our results suggest that teachers are capable of reporting on 3-and 4-year-old children’s communication skills and teacher ratings are a good source of information about children’s language skills. Both parent and teacher reports were congruent with direct assessments. Differently from parents, teachers have the privilege to observe and compare language skills of many same-age children (Vagh et al., 2009). Despite of this, investigating the validity of reports made by parents and teachers against the direct measure of child language comprehension and production, parents turned to be better reporters than teachers.

### 4.2. Mother and teacher reports as predictors of later language skills

Our second aim was to find out how well two different sources of information—parent and teacher reports—predict future language abilities, considering also mother’s education, child gender and reported language difficulties. Results revealed that parental reports on children’s earlier language skills (ECDI-III total scores) and teacher reports were important predictors of language comprehension and production scores assessed by standardized language measures 1 year later. At the same time, the ECDI-III total score predicted later language skills, especially comprehension scores, better than teacher reports. Thus, parental report measure showed in addition to concurrent validity also good predictive validity. This is consistent with previous findings of the validity of CDIs, suggesting that parents are well-informed about their children’s communicative skills (Pan et al., 2004; Fenson et al., 2007).

Furthermore, although inclusion of mothers’ education in the models already containing parent-and teacher-reported language skills at age 3;0 explained variance in future comprehension and production scores significantly better than previous language skills alone, education turned out to be a nonsignificant predictor. The finding did not confirm the effect of parental education on children’s language skills, although this has been frequently reported in the literature (Fenson et al., 2007). Furthermore, subsequent language skills were not predicted by gender. These findings contradict several previous studies reporting gender-differences in language development (Bornstein et al., 2004; Eriksson et al., 2012). A possible explanation may be the relatively high educational level of mothers who participated in our study, as gender differences in language development have been found to be larger in lower SES compared to upper SES families (Barbu et al., 2015).

Most previous research on predictors of language skills has focused more on expressive vocabulary than on other dimensions of infants’ and toddlers’ communicative abilities measured by the CDIs (Fenson et al., 2007). Some of our findings that differ from previous studies (e.g., no effect of mothers’ education, child gender, and reported language difficulties) can be attributed also to older age of children who participated in our study and that we addressed more general language skills.

The use of parent reports with children older than three has been a concern because their communicative abilities have grown and they spend more time out of their homes, being exposed to various conversational partners and interactional contexts. The practical importance of our study is that it proved that parents of children at 3;0 and 4;0 years of age provide adequate information about their children’s language skills and that they are still best reporters on these skills. There remains a need for more information on how good estimators of children language skills teachers are. The utility and validity of teachers as reporters of child language skills is particularly pressing as teachers should identify children with language problems as early as possible. Significant, although modest correlations between mother and teacher reports albeit teacher ratings based only on 3 items provided evidence for the utility of teacher reports to receive useful information about children’s language skills. The study pointed out that in order to understand whether teacher reports are in accordance with parent reports, it is important to compare their ratings by using the same report instrument (e.g., CDIs). Of course, it is time consuming and a burden for teachers to report on each child in the group of many children. Until now, there is only one CDI study comparing parents and teachers as reporters, but it has been done with bilinguals from lower-SES families (De Houwer et al., 2005).

A limitation of the study is that the predictive validity of reports was not studied over a longer interval than 1 year. Longitudinal studies are needed to find out how well the language skills reported for the age group predict their skills in the long term. Moreover, the usability of parent reports of children’s language skills also needs to be investigated for children over 4;0 years of age.

### 4.3. Conclusion

The current study showed that parent reports on children’s language skills at 3.0 and 4;0 years of age are indicative of concurrent language skills, and valid predictors of subsequent language skills 1 year later. There is a need for more information about how good reporters of children’s language skills teachers are when using an assessment tool such as the CDI-III. The knowledge is useful for practice as might reduce negative consequences of language problems by timely identification and support.

## Data availability statement

The raw data supporting the conclusions of this article will be made available by the authors, without undue reservation.

## Ethics statement

The studies involving human participants were reviewed and approved by the Research Ethics Committee of the University of Tartu. Written informed consent to participate in this study was provided by the participants’ legal guardian/next of kin.

## Author contributions

TT formulated the research questions, supervised the data collection, and wrote the first draft of the manuscript. AS organized the dataset, participated in data analysis and interpretation of the data, and participated in writing the method section of the manuscript. All authors approved the submitted version.

## Funding

This study was supported by the Estonian Research Council (grant number: PRG1761).

## Conflict of interest

The authors declare that the research was conducted in the absence of any commercial or financial relationships that could be construed as a potential conflict of interest.

## Editor’s note

Melita Kovacevic edited the article in collaboration with Maria-Jose Ezeizabarrena. Faculty of Arts, University of the Basque Country UPV/EHU Vitoria/Gasteiz, Spain.

## Publisher’s note

All claims expressed in this article are solely those of the authors and do not necessarily represent those of their affiliated organizations, or those of the publisher, the editors and the reviewers. Any product that may be evaluated in this article, or claim that may be made by its manufacturer, is not guaranteed or endorsed by the publisher.
